# Impact of a smoking ban in hospitality venues on second hand smoke exposure: a comparison of exposure assessment methods

**DOI:** 10.1186/1471-2458-13-536

**Published:** 2013-06-04

**Authors:** Sarah Rajkumar, Cong Khanh Huynh, Georg F Bauer, Susanne Hoffmann, Martin Röösli

**Affiliations:** 1 Swiss Tropical and Public Health Institute, Basel, Switzerland; 2 University of Basel, Basel, Switzerland; 3 Institute for Work and Health, Lausanne, Switzerland; 4 Institute of Social and Preventive Medicine, University of Zürich and Center for Organizational and Occupational Sciences, ETH Zurich, Switzerland

**Keywords:** Smoking ban, Hospitality workers, Second hand smoke, Passive sampler, Prospective study

## Abstract

**Background:**

In May 2010, Switzerland introduced a heterogeneous smoking ban in the hospitality sector. While the law leaves room for exceptions in some cantons, it is comprehensive in others. This longitudinal study uses different measurement methods to examine airborne nicotine levels in hospitality venues and the level of personal exposure of non-smoking hospitality workers before and after implementation of the law.

**Methods:**

Personal exposure to second hand smoke (SHS) was measured by three different methods. We compared a passive sampler called MoNIC (Monitor of NICotine) badge, to salivary cotinine and nicotine concentration as well as questionnaire data. Badges allowed the number of passively smoked cigarettes to be estimated. They were placed at the venues as well as distributed to the participants for personal measurements. To assess personal exposure at work, a time-weighted average of the workplace badge measurements was calculated.

**Results:**

Prior to the ban, smoke-exposed hospitality venues yielded a mean badge value of 4.48 (95%-CI: 3.7 to 5.25; n = 214) cigarette equivalents/day. At follow-up, measurements in venues that had implemented a smoking ban significantly declined to an average of 0.31 (0.17 to 0.45; n = 37) (p = 0.001). Personal badge measurements also significantly decreased from an average of 2.18 (1.31-3.05 n = 53) to 0.25 (0.13-0.36; n = 41) (p = 0.001). Spearman rank correlations between badge exposure measures and salivary measures were small to moderate (0.3 at maximum).

**Conclusions:**

Nicotine levels significantly decreased in all types of hospitality venues after implementation of the smoking ban. In-depth analyses demonstrated that a time-weighted average of the workplace badge measurements represented typical personal SHS exposure at work more reliably than personal exposure measures such as salivary cotinine and nicotine.

## Background

Banning smoking at workplaces and restaurants is widely recommended as a key intervention for protecting people from exposure to second hand smoke (SHS) [1-3]. Although the hospitality sector had been previously excluded from smoking bans, this omission has been amended in many countries over the past 10 years. Today, 28 countries have comprehensive policies banning smoking in all workplaces [4]. This trend is in alignment with the recommendations from the World Health Organization’s Framework Convention for Tobacco Control (FCTC), stating in Article 8 that all workplaces in closed rooms should be protected from SHS [5]. Although Switzerland signed the WHO Convention in 2004, it was never ratified.

There is no comprehensive smoking ban protecting hospitality staff from SHS in Switzerland. In May 2010, a national smoking ban based on a fairly unrestricted regulation which permitted certain exceptions was implemented [6]. According to the national law, venues could allow smoking if they were less than 80 m^2^ in size or if smoking rooms did not exceed one third of the total venue size. Switzerland is divided into 26 administrative zones called cantons, and each was permitted to implement its own stricter legislation on top of the national law. This has resulted in a patchwork of different laws within a small geographical area.

It has been shown that a partial law can actually lead to an increase in SHS levels in venues that continue to allow smoking [7]. In a global cross-sectional study measuring smoking and non-smoking Irish pubs, Connolly et al. found particulate matter less than 2.5 μm in diameter (PM_2.5_) to be 93% lower in smoke-free pubs [8]. According to Villarroel et al., PM_2.5_ levels are five times higher in smoking venues than in non-smoking venues [9]. Interestingly, several studies found that spatial separation of rooms where smoking is allowed does not prevent exposure to environmental tobacco smoke in nearby non-smoking areas. In 2004, Cains et al. found that spatially separated non-smoking rooms had only marginally reduced particulate matter less than 10 μm in diameter (PM_10_) and nicotine air levels when compared to a non- smoking area in direct confluence with a smoking area [10]. A Swiss study showed that PM_2.5_ in non-smoking rooms of venues that allowed smoking elsewhere in the building was more than double the PM_2.5_ levels of completely smoke-free hospitality venues [11]. Several longitudinal studies examined changes in SHS levels before and after introduction of smoking bans. While Semple et al. found an average PM_2.5_ reduction of 86% in Scottish pubs two months after implementation of the law, Lee et al. came up with a slight decrease resulting from a partial law compared to a large decrease from a comprehensive law [12,13]. These effects were reproduced in other places such as Minnesota or Guatemala [14,15].

The present study is part of COSIBAR (Cohort Study on Smoke-Free Interventions in Bars and Restaurants), a longitudinal quasi-experimental study examining exposure of hospitality workers who were non-smokers, and their health status at three different time points before and after implementation of the new law. There are several established methods to determine personal SHS exposure, all of which have their advantages and disadvantages. The most common and simple way is through a questionnaire [16]. Other options include taking biological samples such as urine, saliva, blood or hair. While drawing blood is invasive, both urine and saliva sampling are simple and quick. Commonly the cotinine content is measured, as it is the most specific and sensitive biomarker [17]. A hair sample provides cumulative exposure over time, with the last centimetre of hair usually corresponding to the previous month’s exposure [18], but this method needs to be further refined [19]. In order to determine the SHS exposure within a room, PM_2.5_ levels in air are often used as proxies [11]. In this study, SHS exposure of the participants was determined with three different methods. Firstly, by the MoNIC (Monitor of Nicotine) badge, a passive sampling device, secondly, by salivary samples and thirdly, by a personal interview relating to duration of SHS exposure at work and outside of working hours.

The aim of the present study was to analyse the effect of different smoking regulations on SHS exposure in bars, cafés and restaurants and of non-smoking hospitality workers employed therein. In addition, we aimed to evaluate the different methods of SHS exposure assessment and to determine the most accurate proxy for SHS exposure at work.

## Methods

### Study design

**Figure 1:**
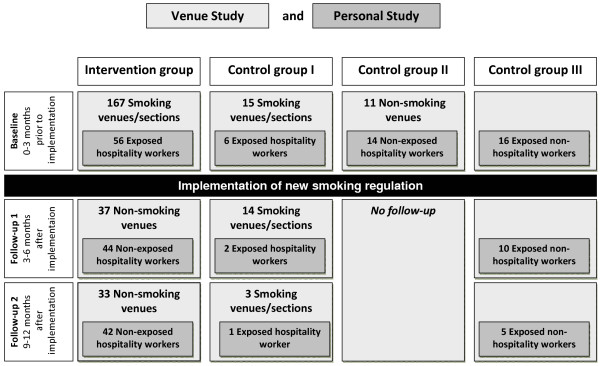
Study design with number of venues and study participants.

This is a quasi-experimental, longitudinal study (Figure 1) comparing two groups: i) hospitality venues and non-smoking employees for whom smoking was banned as a result of a new smoking regulation (intervention group); ii) hospitality venues and non-smoking employees that did not undergo any change in exposure (control groups). The intervention group of the venue study consisted of hospitality venues where smoking was either partially or completely allowed prior to the introduction of the smoking ban. All compliant venues were included in the study. Participants of the personal study had worked in such a venue for at least one year prior to the ban, and were therefore exposed to SHS. Additional eligibility criteria were being between 18 and 65 years of age, working at least half-time and having been a non-smoker for at least 5 years. After introduction of the smoking ban, the intervention group were no longer exposed to SHS at work. The primary comparison group (Control Group I, Figure 1) were employees who were exposed to SHS both before and after the implementation of the smoking ban because of the exceptional rules described above. As hospitality workers who were non-smokers were found to only rarely work in such venues, two additional comparison groups were included in the COSIBAR study. Control Group II consisted of non-smoking hospitality workers who worked in a smoke-free environment at all times, and Control Group III were non-smokers that were regularly exposed to SHS without being employed in the hospitality sector.

In the intervention group, a baseline examination was conducted within the 3 months prior to the introduction of the smoking ban. Subsequently, two follow-up examinations were conducted at 3–6 months and 9–12 months after the smoking ban introduction (Figure 1). Study participants of Control Group II were examined only once for a cross-sectional baseline analysis. Control Groups I and III were examined three times, unrelated to the smoking ban. Most of the study participants worked in the cantons of Basel City, Basel County and Zurich. Smoking bans were introduced on 1^st^ April 2010 in Basel City and on 1^st^ May 2010 in Basel County and Zurich.

### Recruitment procedure, study population and data collection

A list of all hospitality venues in the cantons of Zurich, Basel City and Basel County was created using the digital Swiss phonebook from 2009. Each venue received a letter with information about the study, including a request to distribute screening questionnaires to staff serving at tables or at the bar (waiting staff) and for air measurements to be performed by the study team. These letters were followed with phone calls and visits two weeks later. For those venues that agreed to participate, an interview with the owner was conducted in order to obtain basic information relating to the current smoking regulations, the venue size, other sources of environmental PM_2.5_ and the number of staff. At least one MoNIC badge was placed in the venue, often near the bar where waiting personnel spend much of their working time (hereafter referred to as “workplace badge”). Where there were separate smoking and non-smoking rooms or sections, one badge was placed in each. After one week, badges were collected and evaluated. For the intervention group, air measurements were performed in 167 hospitality venues that allowed smoking at least partly before implementation of the national smoking ban in May 2010. Follow-up measurements were only conducted in those venues where personal study participants worked. The 26 control group venues were recruited and measured by the same procedures between 2010 and 2011.

For the personal study, screening questionnaires were distributed to the waiting staff, providing information on age, workload (hours/week), number of years worked, smoking status, current health and personal details. Eligible study participants were invited to a health examination, which was carried out in one of two study centres in Basel City and Zurich. Prior to the visit, a MoNIC badge was sent to the study participants which they were asked to attach near their shirt collar for a period of 24 hours and bring to the study centre (hereafter referred to as “personal badge”). A protocol stating the exact measurement time and location accompanied each badge. During the visit at the study centre, saliva was collected for nicotine and cotinine analyses. A questionnaire, part of which was conducted as a face-to-face interview, was completed relating to smoking behaviour and SHS exposure over the previous 12 months at work and outside of work. The participants were asked at the baseline visit and at the first follow-up visit how many hours per day they were exposed to SHS at work and during their leisure time. Categorical responses were required for both questions: 0–0.5 hours, 0.5-2 hours, 2–5 hours and more than 5 hours.

Members of Control Group III were recruited by means of an online advertisement looking for non-smokers that were exposed to SHS on a regular basis, either privately or at work. In this group, no workplace badge data was collected.

Ethical approval was obtained from the EKBB (Ethics committee of both cantons of Basel) and all participants signed an informed consent before every examination (Reference no. EK: 317/09).

### Laboratory analyses

MoNIC badges are glass fibre filters that are washed with distilled water, methanol and dichloromethane (CH_2_Cl_2_)_,_ impregnated with about 5 mg sodium bisulphate per filter and placed in an air-tight plastic case [20]. This method was developed by the Institute of Work and Health in Lausanne [20] and adapted from that proposed by Hammond and Ogden [21,22]. Badges were always transported between study centres, participants, and the laboratory in these air-tight cases. The amount of nicotine on the badge was determined by gas chromatography. The extracted nicotine from the filter, known to take in air at a rate of 10 ml/min, was multiplied by 1000 to mimic an average respiration rate of 10 l/min which corresponds to normal sedentary behaviour [23]. The number of passively smoked cigarettes was calculated by assuming 0.2 mg nicotine per cigarette.

Salivary cotinine and nicotine levels were obtained during the medical examination without any stimulation using a plastic straw, and quantified by liquid-liquid (liq-liq)extraction with CH_2_Cl_2_ and GC-NPD (gas chromatography-nitrogen-phosphorus detector; working range: 0.1-500 ng/ml. limit of quantification: 0.1 ng/ml) [20,24]. The final batch of saliva samples from control group members were excluded from the data analysis due to inconsistencies in lab procedures.

### Data analysis

Data were analysed using Stata 10.1. Workplace exposure of participants was calculated from the MoNIC badge placed at the workplace. If two badges were placed in a venue, we used the value from the section where the buffet was located for our calculations as waiting staff spends more of their time there. We used a time-weighted average workplace exposure taking into account work load and regular work time. The badge value was multiplied by 1.75 considering that nicotine levels decrease when the venue is closed which is about 72 hours/week corresponding to eight hours/day and one full holiday/week. For a full-time employee this number was divided by three to calculate cigarette equivalents (CE)/day assuming an eight hour shift/day. This factor was estimated from a previous study of continuous PM_2.5_ measurements in smoking environments [11]. Longitudinal comparisons were conducted by means of the Kruskal-Wallis equality-of-populations rank test that compares three or more unmatched groups. In cross-sectional comparisons between different venue types, the non-parametric Mann–Whitney test for two unpaired groups was applied.

## Results

**Figure 2:**
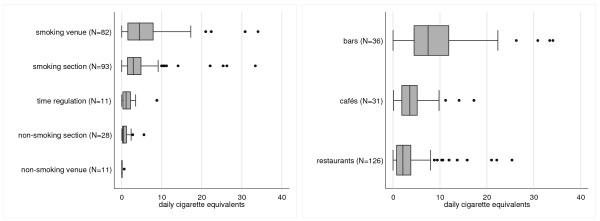
**Cross-sectional comparison of SHS levels in different types of hospitality venues (n = 193) with different smoking policies at baseline (n = 225). **^1^ Time regulation refers to venues with temporal ban, ex. during mealtimes. ^2^ A Cigarette equivalent (CE)/day is the equivalent amount of smoke of one actively smoked cigarette that a smoke-exposed person inhales.

We performed 225 badge measurements in 193 hospitality venues during baseline visits. First follow-up visits were conducted 199 days later, on average, in 51 venues with 58 badges. At the second follow-up, 42 badges were placed in 36 venues. The intervention group comprised 56 persons at baseline, 44 persons at follow-up 1 (79%) and 42 at follow-up 2 (75%). These were compared to the control groups: 6 persons working in smoking venues that did not change their rule (control group I), 14 hospitality workers that had always worked in smoke-free environments (Control Group II) and 16 persons that are regularly exposed to SHS privately or at work without being employed in the hospitality sector (Control Group III). Two members of Control Group I returned for a second examination (33%), while 10 (62.5%) and 5 (31.3%) participants in Control Group III underwent second and third examinations, respectively (Figure 2).

At baseline, badge analysis of all smoke-exposed hospitality venues yielded an average value of 4.48 (95%-CI: 3.7 to 5.25; n = 214) CE/day. This means that a person present in a smoke-exposed venue for 24 hours would inhale a similar amount of smoke as a person actively smoking 4.48 cigarettes. Badges from smoking venues from where at least one study participant was included in the study (n = 50), yielded an average exposure of 4.00 (95%-CI: 2.48 to 5.51) CE/day. The other 164 badges from venues without study participants had average values of 4.62 (95%-CI: 3.72 to 5.53) CE/day. The type of smoking regulation in place clearly influenced SHS exposure, with smoking venues reaching an average of 6.12 (4.71 to 7.53; n = 82) CE/day (Figure 2). Levels in smoking sections yielded 4.39 (3.24 to 5.54; n = 93) CE/day, venues with a time regulation that prohibited smoking during mealtimes 1.95 (0.45 to 3.45; n = 11) CE/day and open, non-smoking sections averaged 0.92 (0.5 to 1.35; n = 28) CE/day. This was still significantly higher than values in completely non-smoking venues (Control Group II) at 0.13 (0.01 to 0.24; n = 11) CE/day (p < 0.001). There was only one non-smoking section in our sample that was separated by a door.

There were also substantial differences in SHS exposure according to the type of hospitality venue. At baseline, the highest exposure was found in bars with 9.99 (7.06-12.92; n = 36) CE/day, followed by cafés (4.54; 95% CI: 3.12-5.96; n = 31) and restaurants (3.28; 2.53-4.02; n = 126) (Figure 2).

At the time of the first follow-up, the exposure had significantly decreased to 0.3 (0.21 to 0.38; n = 37) in all venues that had introduced a smoking ban (i.e. intervention group), irrespective of venue type (p < 0.001). The type of venue was no longer an influential factor. Six months later, at follow-up 2, levels remained low at 0.33 (0.17 to 0.49; n = 33) CE/day. In venues that had not implemented a smoking ban (Control Group I), smoke levels at follow up 1 were, on average, 3.37 (1.29 to 5.44; n = 14) CE/day.

Table 1 shows the results of the personal study. At follow-up, personal badges showed significant decreases in exposure (p < 0.001), although this decrease was not as pronounced as for workplace badges. Restricting the pre/post ban comparison of the intervention group to only the 33 persons who participated in all three examinations gave similar results (mean time-weighted average of workplace exposure at baseline: 2.67 (1.38 to 3.96) CE/day, at first follow-up: 0.14 (0.1 to 0.18) CE/day, and at second follow up: 0.19 (0.09 to 0.28) CE/day). Participants that were lost to follow-up had lower workplace exposure at baseline (p = 0.004), in the personal badges this difference was less pronounced (p = 0.171). In the questionnaire, 14.7% of the intervention group reported the same length of exposure per day at follow-up as at baseline, while the remainder reported lower values (85.3%). Regarding exposure in their leisure time, 30.3% stated a lower number of exposed hours, 66.7% remained the same and one person reported an increase in exposed hours.

**Table 1:** Different personal measurement methods at baseline, follow-up 1 and follow-up 2

		**Baseline**		**Follow-up 1**		**Follow-up 2**		
		**n**	**Arithmetic mean (95% CI)**	**n**	**Arithmetic mean (95% CI)**	**n**	**Arithmetic mean (95% CI)**	**p-Value**
Personal badge (cigarette equivalents/day)	Intervention group	**53**	**2.18 (1.31 to 3.05)**	**41**	**0.53 (0.39 to 0.67)**	**41**	**0.25 (0.13 to 0.36)**	**<0.001**
	Control Groups I + III	**20**	**1.69 (0.74 to 2.64)**	**11**	**1.17 (0.57 to 1.77)**	**5**	**1.22 (0.10 to 2.34)**	**0.892**
	Control Group II	**14**	**0.41 (0.25 to 0.57)**					
Work place badge (cigarette equivalents/day)*	Intervention group	**50**	**4.82 (3.14 to 6.50)**	**40**	**0.27 (0.19 to 0.34)**	**36**	**0.31 (0.17 to 0.46)**	**<0.001**
	Control Group I	**6**	**3.85 (−0.51 to 8.21)**	**2**	**2.15 (−23.66 to 27.96)**	**1**	**0.22**	**0.561**
	Control Group II	**12**	**0.27 (0.08 to 0.47)**					
Time weighted average of work badge (cigarette equivalents/day)*	Intervention group	**50**	**2.65 (1.69 to 3.62)**	**40**	**0.15 (0.11 to 0.20)**	**36**	**0.18 (0.10 to 0.27)**	**<0.001**
	Control Group I	**6**	**2.24 (−0.30 to 4.79)**	**2**	**1.25 (−13.80 to 16.31)**	**1**	**0.30**	**0.561**
	Control Group II	**12**	**0.14 (0.03 to 0.25)**					
Salivary nicotine (ng/ml)	Intervention group	**44**	**1.99 (0.98 to 3.00)**	**35**	**2.42 (−0.01 to 4.86)**	**36**	**2.81 (−0.12 to 5.75)**	**0.227**
	Control Groups I + III	**14**	**1.45 (−0.05 to 2.95)**	**5**	**4.24 (−5.49 to 13.97)**	**1**	**7.80**	**0.699**
	Control Group II	**5**	**2.38 (−3.95 to 8.71)**					
Salivary cotinine (ng/ml)	Intervention group	**44**	**0.67 (0.04 to 1.30)**	**35**	**2.75 (0.32 to 5.17)**	**36**	**0.81 (0.00 to 1.61)**	**0.243**
	Control Groups I + III	**14**	**1.54 (−0.43 to 3.51)**	**5**	**3.84 (−2.56 to 10.24)**	**1**	**0.13**	**0.329**
	Control Group II	**5**	**2.82 (−4.73 to 10.37)**					

*no data for control group III.

^1^if two persons worked in the same venue, this badge was counted double.

Addressing the second aim of the study, the personal badge results were compared with salivary nicotine and cotinine levels. Spearman’s rank correlation coefficients were 0.17 for badge versus nicotine (p-value: 0.04; n = 137) and 0.3 for badge versus cotinine (p-value < 0.001; n = 137). Nicotine and cotinine showed a correlation of 0.41 (p-value: <0.001; n = 140). The time-weighted average of the workplace badge yielded correlation coefficients of 0.17 with salivary nicotine (p-value: 0.07; n = 116), 0.23 with salivary cotinine (p-value: 0.01; n = 116) and 0.56 with the personal badge (p-value < 0.001; n = 142).

## Discussion

The smoking ban led to a significant decrease in exposure for all participants that worked in an environment where a new law was introduced. SHS levels in all types of venues dropped to nearly zero after the ban. At baseline, the current smoking policy in the venue and the type of venue clearly influenced the number of passively smoked cigarettes per day, as calculated from air measurements in hospitality venues. Venues where participants worked did not significantly differ from venues from where there were no participants. Bars had higher values than cafés, which in turn yielded a higher exposure than restaurants. Some restaurants had special time regulations such as smoke-free mealtimes, which led to a further decrease in average levels. Badge readings from smoking rooms were only slightly higher than those from smoking sections in mixed rooms, i.e. rooms containing both smoking and non-smoking sections. Although non-smoking sections had lower levels, they were, nevertheless, significantly higher than in entirely smoke-free locations. These results are in line with previous studies that found that designated non-smoking sections were inadequate measures for protecting people from SHS [10,11,25]. Moreover, studies have shown that only the implementation of a comprehensive law results in widespread acceptance by the population [26,27].

### Comparison of various exposure proxies

In this study, salivary data were compared with personal and workplace badge measurements as well as questionnaire data. There was poor correlation between these indicators. The personal and the workplace badges had the highest correlation coefficient: 0.56 (p-value < 0.001; n = 142). One drawback of personal badge measurements is that participants did not always clearly state where the badge was worn, be it at home, work or both. The personal badge measurement was also greatly affected by the behaviour on that given day, e.g. whether the wearer went out to a place where smoking was allowed. In some instances, the personal badges captured only exposure outside of the workplace, as some study participants did not work during the 24 hours prior to the health examination. The results of these badges are, therefore, specific to a given day and should be treated with caution. The workplace badge, however, was exposed for a period of one week, thus representing the average exposure environment in the workplace.

Salivary measurements are subject to variations in individual metabolism, as are all biomarkers. Cotinine reflects exposure to nicotine and is, therefore, very specific for tobacco exposure [28]. Timely sampling is crucial, due to the rapid degradation of the compound, and results also depend on how recently the exposure occurred [29]. They would have been more likely to reflect workplace SHS exposure if participants were sampled immediately after leaving the workplace. Unfortunately, this was not possible in the context of this study. But other studies face similar problems, in particular if the exposure is not as clearly specified as in hospitality workers.

Our salivary samples do, however, allow us to identify potential smokers from a supposedly non-smoking sample. The Society for Research on Nicotine and Tobacco (SRNT) subcommittee suggests that smokers are likely to have salivary cotinine values of > 15 ng/ml [30]. Thus, salivary measurements will be useful to ensure that future health analyses are restricted to non-smokers only. Self-reported data in questionnaires are prone to recall bias and risk imprecision [28]. Nevertheless, participants confirmed their overall declining exposure at work with the responses given in the questionnaire. Concerning exposure out of working hours, a declining tendency was observed, but two thirds reported unchanging conditions.

We calculated a time-weighted average for the workplace badge to better approximate the true exposure at work, taking into account the participants’ individual workload. For this reason, workplace badges likely provided the most relevant measure of changes in exposure after the smoking ban introduction. Consequently, these measurements were used to assess the relationship between changes in workplace exposure and cardio respiratory outcomes.

### Strengths and limitations

A key strength of our study is that different methods were applied and compared in order to assess the personal exposure of the study participants. In addition to the more standard methods of salivary nicotine and cotinine and questionnaire-based data, the MoNIC badge was used. This passive sampler is very simple and easy to use, providing a specific value for nicotine without a proxy. Although SHS is the most important contributor, PM_2.5_ measurements can be confounded by other sources such as candles, kitchen fumes or other air pollutants [31,32]. A large number of venues were measured before the smoking ban, representing a range of venue types and smoking policies. Some limitations to our study have been identified. The recruitment process was long, taking place both before and after implementation of the new law, and this could have led to a selection bias. Presumably, venues less willing to participate would have had higher exposure levels. Restaurants were overrepresented in our study compared to bars, and restaurants had lower SHS levels than bars. Consequently, an underestimation of the true average SHS levels in Swiss hospitality workers is highly likely. A similar situation can be assumed in the recruitment of participants. Heavily exposed workers were often determined to be smokers, and were not eligible to participate. Those workers that consented to participate were probably more health conscious and more likely to work in venues with lower exposures.

## Conclusions

These results support previous findings that a smoking ban leads to significantly lower SHS levels in hospitality venues, provided that the venue is completely smoke-free. A time-weighted average of the workplace badge turned out to be the most reliable method to determine changes in personal SHS exposure at the workplace. The personal exposure of hospitality workers was shown to decline after the implementation of a smoking ban. A comprehensive national law is needed in hospitality venues in Switzerland in order to fully protect the population from SHS, particularly hospitality personnel in their professional environment.

## Competing interests

The authors declare that they have no competing interests.

## Authors’ contributions

SR conducted the study, analysed the data and drafted the manuscript. CKH contributed to the design of the study, performed the laboratory analysis and revised the manuscript. GFB contributed to the design of the study and revised the manuscript. SH designed a questionnaire and revised the manuscript. MR designed the study, analysed the data and revised the manuscript. All authors read and approved the final manuscript.

## Pre-publication history

The pre-publication history for this paper can be accessed here:

http://www.biomedcentral.com/1471-2458/13/536/prepub
